# Using Concept Mapping to Identify Community Partners’ and Researchers’ Perceptions of Social Justice: A Path Toward Eliminating Chronic Disease Disparities

**DOI:** 10.1089/heq.2023.0230

**Published:** 2024-06-27

**Authors:** Eric K. Soule, Dina M. Jones, Nakita Lovelady, Luke Thomas, Ruofei Du, Theresa E. Prewitt, Elizabeth Taylor, Sydney Baker, Mignonne C. Guy, Carol E. Cornell, Pebbles Fagan

**Affiliations:** ^1^ Department of Health Education and Promotion, East Carolina University, Greenville, North Carolina, USA.; ^2^ Fay W. Boozman College of Public Health, University of Arkansas for Medical Sciences, Little Rock, Arkansas, USA.; ^3^ Department of African American Studies, Virginia Commonwealth University, Richmond, Virginia, USA.

**Keywords:** social justice, disparities, health equity, cancer, cardiovascular disease, concept mapping, community engagement

## Abstract

**Background:**

A social justice framework can be used to inform healthy equity-focused research, and operationalizing social justice can inform strategic planning for research and practice models. This study aimed to develop a working definition of social justice based on input from a diverse group of collaborators to better inform the work conducted within the Center for Research, Health, and Social Justice.

**Methods:**

A concept mapping study was conducted from March to May 2022. A prompt designed to elicit social justice themes was developed (phase 1). At a study website, participants brainstormed statements that represented their definition of social justice (phase 2). Participants then sorted statements based on similarity and rated statements on importance (phase 3). Multidimensional scaling and hierarchical cluster analysis were used to identify nonoverlapping thematic clusters of statements (phase 4). Models were reviewed for best fit, and clusters were assigned names based on theme (phase 5).

**Results:**

Participants (*n* = 49) generated 52 unique statements that were sorted into 5 clusters describing social justice themes. Clusters included (1) Empathy, Awareness, and Understanding (*n* = 11); (2) Education and Systems Change (*n* = 10); (3) Policy Design and Implementation (*n* = 9); (4) Equity and Leveling the Playing Field (*n* = 11); and (5) Access to Services and Fair Living Standard (*n* = 11). High mean cluster ratings ranging from 5.22 to 6.02 out of 7 indicated all clusters were rated as being very important aspects of social justice.

**Conclusions:**

These data can guide the restructuring of research ecosystems that help eliminate race- and place-based health disparities.

## Introduction

In 2021, the National Institute on Minority Health and Health Disparities (NIMHD) awarded over $200 million in funds to “establish and support regional comprehensive research centers on the prevention, treatment, and management of comorbid chronic diseases that disproportionately affect populations with health disparities.”^1^ The Center for Research, Health, and Social Justice is one of 11 Multiple Chronic Disease Centers funded to address determinants of health at multiple levels.^2^ The Center goals are to (1) advance the science of chronic disease health disparities by using transdisciplinary, multilevel, and social structural approaches; (2) facilitate research and training opportunities to strengthen the capacity of researchers and community members to develop interventions; and (3) support equitable academic–community partnerships to address the root causes of chronic disease health disparities and promote sustainable solutions for Black/African American people and people living in rural communities.

The Center was established with an underlying value that social justice approaches are needed to address the multilevel determinants embedded in the Minority Health and Health Disparities Framework.^2^ Determinants of health include the conditions in which people are born, learn, work, worship, play, and age.^3^ Minoritized groups, such as Black/African American people, irrespective of their backgrounds, experience social and structural determinants across their lifespan that can result in cancer and cardiovascular disease disparities. Cancer and cardiovascular disease mortality rates rank highest in the southern region of the United States^4,5^ and Black/African American people,^5–7^ more than 58% of who live in the South.^8,9^ Residents in rural areas, particularly in the South, experience higher cancer^7^ and cardiovascular disease mortality rates than urban residents.^10^

Social justice approaches in research, education, and practice are not new.^11–16^ Powers and Faden^17^ stated that the “moral justification for the social institution of public health is social justice.” These pioneer researchers and organizations, such as the American Public Health Association,^18^ were later joined by others who embrace the importance of social justice approaches in research following the televised murder of George Floyd in May 2020 and the onset of the COVID-19 pandemic. The open acknowledgment of the importance of social justice approaches has increased among researchers,^19–21^ professional organizations,^22–24^ and funding agencies.^25,26^ Growing acknowledgment that social and structural factors are underlying causes of chronic disease health disparities^27^ has also provided impetus for the advancement of social justice approaches.

Prior studies examined the literature to develop a comprehensive definition of social justice to inform nursing practices.^13,28^ To our knowledge, studies have not used a robust participatory approach that includes researchers, practitioners, and community partners to guide the development of a comprehensive definition and social justice domains. Social justice is multifaceted,^29^ and its meaning may depend on lived experience, social status, geography, and other contextual factors. For example, Black/African American people may view social justice differently than those who have historically benefited from the systemic oppression that occurred via colonialism, imperialism, commercialism, racism, and discrimination. Social justice is an approach that can drive operations, research, relationships, training, community engagement, and practices to reduce disparities. This study’s purpose was to understand diverse perceptions of the meaning of social justice to inform strategic planning for research and practice models used in the Center for Research, Health, and Social Justice.

## Methods

The Center for Research, Health, and Social Justice leverages the expertise of key academic partners across multiple universities including the University of Arkansas for Medical Sciences, Virginia Commonwealth University, and East Carolina University; partnerships with the consortium of Center for Multiple Chronic Diseases and Disparities^30^; community partners; professional organizations; partners in research and training implementation; and partners who participate in community outreach and engagement. In 2022, the administrative core of the Center used concept mapping to operationalize the meaning of social justice. In the late 1980s, Trochim developed concept mapping as a structured conceptualization approach to inform the development of frameworks for planning and evaluation.^31^ Concept mapping is defined as a participatory mixed-methods research approach that solicits ideas from a target group in response to a focus prompt^31^ and includes the generation of domains, structuring of the domains, and representation of those domains in the form of a map.

The six concept mapping phases are (1) preparation and development of a focus prompt and selection of participants from the audience of interest, (2) generation of ideas through brainstorming by the audience, (3) structuring of statements including sorting and rating by the audience, (4) representation of statements using quantitative analysis, (5) interpretation through identifying and naming thematic clusters by the audience, and (6) utilization of maps for planning and evaluation. This article reports on phases 1–5. The current study was participatory in that we sought feedback from academic researchers and partners affiliated with our Center to develop the focus prompt, asked a diverse participant sample to respond to an open-ended prompt and sort and rate statements to help establish a social justice framework, and worked with our social justice team, which includes a diverse group of members affiliated with the Center, to finalize statements and the social justice model. Lastly, we presented the results to participants of the community health impact conference, *Social Justice in Turbulent Times: Reclaiming Our Space*, in September 2022 to obtain feedback and input on the findings from community members. This is described further below. This study was approved by the University and Medical Center Institutional Review Board at East Carolina University.

### Phase 1. Preparation of prompt and selection of participants

Eligible participants included the Center’s academic partners from majority- and minority-serving institutions, directors of Center cores, staff, organizational representatives, community partners, and advisory board members. Participants were representative of the groups that are the focus of the Center studies: Black/African American individuals, rural residents, and people who have a history of working with these populations in Arkansas (*n* = 109). The multiple principal investigators and the co-investigator who managed the current concept mapping study were ineligible to participate. The Center’s Measures, Evaluation, and Methods team, which includes principal investigators of the Centers' research projects, community health workers, staff, and co-investigators, discussed and finalized a focus prompt: “Something specific that you think represents or is meant by the term ‘social justice,’ or a specific action, behavior, or strategy that can be taken to promote social justice is…” We chose a broad focus prompt to solicit a wide range of responses from the participant pool, which was diverse regarding age, race, ethnicity, profession, and lived experiences. A study website also provided guidance on the purpose of the study, which was as follows: “The purpose of this study is to gain a better understanding of social justice in order to inform strategies to help prevent chronic disease in Arkansas using a social justice framework. To help us examine this issue, we want you to think about what social justice means to you. Social justice may relate to many things including ideas, behaviors, policies, beliefs, or other issues.”

### Phase 2. Brainstorming ideas

In March 2022, research staff sent email invitations to eligible individuals including a brief description of the study. Interested individuals followed a link to the study website (The Concept Systems^®^ Global MAX™) where they completed an online consent form. Following completion of the consent form, participants were instructed to complete a brief questionnaire that assessed participants’ occupation, years in occupation, type of workplace, age, gender and sex identity, race, and ethnicity. Participants then completed a brainstorming exercise in which each participant provided statements that completed the focus prompt. Participants were allowed to provide multiple statements, and no minimum or maximum number of statements was required. Submitted statements were uploaded to a combined list at the study website. Each statement was anonymous in that there were no means to link a specific statement to the participant who submitted the statement. Each participant completed this task individually; however, statements that had been entered by previous participants were visible in the brainstorming task by participants who completed the task later. This process can generate more ideas because of not having to wait one’s turn to provide a response and the ability to review others’ responses.^32,33^ Participants were instructed to review the list of statements and attempt to avoid submitting statements that duplicated content that had been submitted previously, consistent with previous concept mapping studies.^34–37^ In total, 49 participants completed the brainstorming task and generated 83 statements. Participants received a $10 e-gift card for brainstorming completion.

Four researchers reviewed the statement list independently to remove statements unrelated to the focus prompt and redundant statements. The team came to consensus on the statements to be removed and retained through group discussion. After review, a final list of 52 statements was identified and uploaded to the study website.

### Phase 3. Structuring: Sorting and rating statements

As part of the participatory nature of concept mapping, the final model, which includes a visual display of content themes, is determined based on stakeholders’ organization of the statements. Therefore, in a separate exercise that occurred approximately 1 month after brainstorming, participants who completed the brainstorming activity (*n* = 49) were asked to sort the 52 statements into groups of similar content or theme and rate each statement on importance related to social justice. Of the 49 participants who were invited to complete the sorting and rating tasks, 22 completed the sorting task and 20 completed the rating task. In the sorting task, participants were asked to organize each statement from the list into “piles” of statements based on content similarity.^38,39^ After sorting all statements into piles, participants were asked to rate each statement based on the prompt, “This is something that I feel is an important aspect of social justice,” using a scale (definitely NOT important for social justice = 1 to Definitely important for social justice = 7). Participants received a $25 e-gift card for completing the sorting task and a $10 e-gift card for completing the rating task.

### Phase 4. Representation

**FIG. 1.**
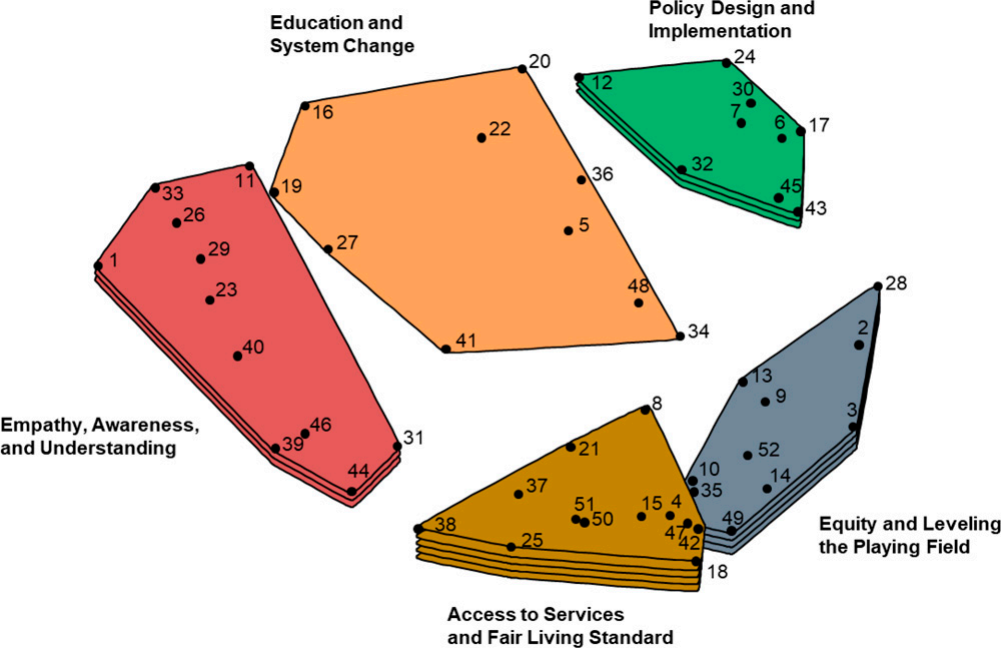
Cluster map of social justice themes. Numbered points on the map correspond to numbered statements displayed in Table 2. Greater number of layers represent clusters with higher mean statement ratings.

After participants completed the sorting and rating tasks, participant sorting data were analyzed using nonmetric multidimensional scaling. Using an algorithm,^40^ each statement was assigned a coordinate (x,y) in two-dimensional space with each statement being represented by a point on a “point map” (see Fig. 1). Points on the map that were closer to each other represented statements that were sorted together more often by participants. The stress value of the model (0.27), which indicates the fit of the multidimensional scaling analysis, fell within the range of stress values reported in previous research,^41^ indicating good fit and congruence between the processed data and the raw data.^40,42^

Clusters of statements related to similar content were identified empirically using a hierarchical cluster analysis in which an algorithm identified nonoverlapping cluster arrangements.^43^ This analysis identifies “clusters” by identifying groups of statements that minimizes the distance between the statements and the centroid of identified clusters, starting with a two-cluster model. Subsequent models were built from the original two-cluster model by separating one cluster into two clusters using the same algorithm. This process was repeated until several models were considered ranging from 5 to 8 clusters.

### Phase 5. Interpretation

Seven team members with experience in social justice research identified the best-fitting model using interpretability (i.e., each cluster described a single idea) and parsimony (i.e., model with fewest clusters preferred) as indicators of best model fit. A final five-cluster model was determined to be the best-fitting model, and the research team assigned names to each cluster based on statement content. Mean ratings were calculated for individual statements and for all statements within each cluster. An *ad hoc* social justice working group, which included concept mapping study participants, reviewed and revised the wording of the final statements and clusters for clarity.

## Results

Table 1 displays characteristics of participants in the sample who completed the brainstorming exercise (*n* = 49). The average age of participants was 52.2 (standard deviation [SD] = 14.2) and approximately two-thirds (67.4%) identified as women. The majority identified as either Black or African American (51.0%) and White or European American (40.8%). Multiple professions were represented including academic researchers (34.7%), community advocates (12.2%), community organization administrators (8.2%), public health practitioners (6.1%), and community health workers (6.1%). Participants reported an average of 18.7 years (SD = 13.0) in their professions.

**Table 1.** Participant Characteristics (*n* = 49)

	N	%
Age (M [SD])	52.2 (14.2)	
Gender		
Woman	33	67.4
Man	15	30.6
Transgender woman	0	0
Transgender man	0	0
Non-binary or gender nonconforming	0	0
I prefer not to answer	1	2.0
Race		
American Indian/Alaskan Native	0	0
Asian	0	0
Native Hawaiian/Pacific Islander	0	0
Black/African American	25	51.0
White/European American	20	40.8
More than one race	2	4.1
Other race	1	2.0
I prefer not to answer	1	2.0
Ethnicity		
Hispanic or Latino(a)	0	0
Not Hispanic or Latino(a)	48	98.0
Prefer not to answer	1	2.0
Profession		
Academic researcher	17	34.7
Academic teacher	1	2.0
Advocate	6	12.2
Administrator in community organization	4	8.2
University or college administrator	8	16.3
Public health practitioner	3	6.1
Community health worker	3	6.1
Other	7	14.3
Time spent in profession (M [SD])	18.7 (13.0)	
Workplace environment		
Academic	26	53.1
Public health	13	26.5
Social service	3	6.1
Human service	1	2.0
Other	6	12.2

SD, standard deviation.

**Table 2.** Clusters and Statements Describing What is Meant by and Examples of Social Justice.

Cluster	Statement	Average rating
Empathy, awareness, and understanding		5.70
	39. Dedication to ensuring race, religion, and income are not indicators of life expectancy.	6.30
	44. True inclusion and access to improve the lives of all people, especially in our vulnerable populations and communities.	6.30
	29. Requires careful listening and understanding regarding all the influences on another person’s life.	6.05
	11. Acknowledgment of the explicit and implicit oppression of socially disadvantaged communities.	5.90
	31. Valuing all people and communities equally.	5.85
	23. Valuing others and treating them with dignity and love.	5.80
	46. Living in a society where people of color are judged as individuals, not as a group, the same way people in the dominant group are judged.	5.68
	1. Becoming cognizant of our own contributions to social injustices.	5.65
	40. Understanding the needs of your service areas and providing an equitable solution to a problem.	5.35
	26. Making sure to not shame or blame people who do not fully understand the concept of social justice.	5.15
	33. There is not any conscious or unconscious internalization of the belief system that everything produced by the dominant group in society is automatically better.	4.70
Education and systems change		5.22
	41. Providing protection to those who are vulnerable.	6.00
	34. The ability to vote without prejudice.	5.85
	22. Ensuring the U.S. education system tells the full story of the American experience as lived by minoritized and majority populations.	5.75
	27. Taking responsibility for the welfare of all residents of the state and not just one’s own welfare.	5.37
	16. Requires a lot of grassroots hours to ensure the intended community can trust and relate to frameworks designed to meet a shared and desired outcome.	5.15
	5. Changing an unfair culture and belief system through kindness and education.	5.10
	20. Righting historical and ongoing wrongs to promote equal and just social, political, and financial opportunities for all.	5.05
	48. The advancement of society to establish and maintain equality for disadvantaged communities compared with cisgender heterosexual White men.	4.89
	36. Providing reparations to those who have been wronged.	4.65
	19. The dominant group in authority operates from the center of respect and has the full trust of nondominant groups to manage monetary funds, large or small.	4.32
Policy design and implementation		5.71
	45. Being intentional in the design of equitable policies, systems, structures, and practices by which our society functions.	6.53
	6. Changes in policy and actions to reframe and restructure systems that have historically oppressed minoritized groups.	6.37
	43. Removing structural and community-based barriers that have disparaged individuals or groups based on social constructs and then creating and maintaining equitable resolutions.	6.25
	17. Changing the policies and systems that cause a need rather than just addressing the need itself (e.g., not only giving a hungry person food, changing the policies that cause them to need food).	6.20
	30. Not only gaining insight from various demographic groups, but also making policy change from the perspective of all.	5.90
	7. Operationalizing the 13th, 14th, and 15th Amendments that outlawed slavery, ensured citizenship regardless of race, and guaranteed the right to vote for all to ensure that all Americans are free from colonizer legacies that keep Americans in perpetual bondage.	5.45
	24. Punishing police brutality more harshly.	5.42
	32. Providing earlier education on financial literacy and credit to promote social justice through passive income.	4.70
	12. Encompassing a new abolitionist mindset and framework to address the inherent structural violence of state-sponsored and state-endorsed acts of omission and commission.	4.60
Equity and leveling the playing field		5.77
	9. Assurance of rights, privileges, and freedoms to all individuals.	6.40
	14. Equal application of rules to everyone regardless of race, color, creed, or socioeconomic status.	6.25
	49. Equal opportunities to everyone regardless of race, color, creed, or socioeconomic status.	6.21
	4. Every individual, regardless of age, race, ethnicity, gender, sexual persuasion, religious affiliation, and level of physical ability is treated with respect, courtesy, and equal opportunity for the pursuit of health and happiness.	6.20
	13. Applying the rules of our society equitably, not just equally.	5.90
	28. Social justice encompasses actions needed to achieve communal/spatial justice, economic justice, educational justice, electoral justice, environmental justice, food justice, and information/media justice.	5.85
	52. When the playing field is level.	5.42
	3. Equitable distribution of wealth, opportunities, and privileges regardless of race, gender, age, sexual orientation, etc.	5.40
	35. Equality in society for socially disadvantaged groups as well as dominant and non-disadvantaged groups.	5.37
	10. Each person equitably experiences the benefits and the costs of living in society.	5.30
	2. Removal of privilege from one or more dominant groups and the equal redistribution of that privilege.	5.10
Access to services and fair living standard		6.04
	42. Individuals are treated equally economically, politically, and socially regardless of race, gender, religion, disability, veteran status, familial status, socioeconomic status, health care, schooling, housing, etc.	6.55
	47. Ensuring access to the same quality of services (e.g., education, employment, and health care) for all.	6.17
	37. Equal justice before the law and equitable enforcement of the law without regard to race, ethnicity, gender, religion, or social class.	6.15
	15. Ensuring equality in access to health care.	6.10
	21. Social justice is participatory and involves everyone having equal opportunities to be heard.	6.10
	51. Everyone can earn a fair and livable wage.	6.00
	18. Ensuring not just equal, but equitable access to education, health care, and opportunity.	6.00
	8. Promoting and maintaining diversity, human rights, equity, access, and inclusion regardless of race, sex, gender, age, sexual orientation, socioeconomic background, disabilities, etc.	6.00
	50. Everyone has the right to fair housing.	5.89
	25. Each individual/family has the opportunity to rise above poverty and thrive.	5.80
	38. All people are able to fulfill their full potential.	5.65

Mean ratings are based on responses to the prompt, “This is something that I feel is an important aspect of social justice,” using the scale 1—“Definitely NOT true for social justice” to 7—“Definitely true for social justice.” Statement numbers correspond to the points in Figure 1.

Figure 1 shows the five clusters that described domains of social justice. These clusters ranged in size from 9 to 11 statements. Mean ratings ranged from 5.22 to 6.04 (out of 7) based on the prompt, “This is something that I feel is an important aspect of social justice.” A description of each cluster is summarized in the next section. See Table 2 for a full list of statements within each cluster.

### Cluster 1: Empathy, awareness, understanding (n = 11)

The overall mean rating was 5.70 (SD = 0.46), and the statements in this cluster were rated on average the highest compared with other clusters. Statements in this cluster emphasized that social justice is being inclusive, judging and valuing people equally, listening carefully to understand factors that influences one’s life, treating people with dignity and love, and making sure that demographics do not determine life expectancy. The statements focused on people being aware of explicit and implicit oppression, one’s own contributions to social injustices, and being aware of conscious or unconscious beliefs about who in society is better than another. The statements also focused on understanding of the needs of people and service areas and reducing blame on people who do not understand social justice.

### Cluster 2: Education and systems change (n = 10)

The overall mean rating of statements in this cluster was 5.2 (SD = 0.51). Statements described how social justice was defined as ensuring that American history tells the truthful and complete stories about the experiences lived by all populations. In addition, statements described the need to change unfair culture and beliefs that have led to disparities by actions such as education, “righting the wrongs,” and protecting vulnerable people.

### Cluster 3: Policy design and implementation (n = 9)

The mean cluster rating was 5.71 (SD = 0.67), and the statements described how social justice encompasses being deliberate about changing and designing policies, systems, and structures in a way to address the root causes of inequities, such as creating policies that promote equitable outcomes and take into consideration multiple perspectives. Statements also described the importance of operationalizing the amendments of the U.S. Constitution, removing structural and community barriers, and punishing policy brutality against people more harshly. Statements described a strategy for promoting social justice through passive income and financial literacy and creating an abolitionist mindset to address structural violence.

### Cluster 4: Equity and leveling the playing field (n = 11)

Statements in this cluster (mean rating= 5.77, SD = 0.44) focused on the idea that social justice is achieved when the playing field is level. This was operationalized in several ways, including equitable distribution of wealth, opportunities, and privileges. Statements indicated that societal rules should benefit and be enjoyed equitably by all as evidenced by statements describing the need for the assurance of rights, freedoms, respect, courtesy, and the ability to pursue happiness and health.

### Cluster 5: Access to services and fair living standard (n = 11)

The overall mean rating of this cluster was 6.04 (SD = 0.22), and the statements described social justice as people having the access to quality and equitable health care, fair housing, education, employment, and the ability to rise above poverty. This was summarized in the statement that had the highest rating of all statements, “Individuals are treated equally economically, politically, and socially regardless of race, gender, religion, disability, veteran status, familial status, socioeconomic status, health care, schooling, housing, etc.” Statements focused on the idea that people should be treated equally, including as part of law enforcement, no matter who they are, and should have the opportunity to fulfill their full potential in life. Statements also emphasized the importance of promoting diversity and inclusion of all people. One statement reflected the idea that social justice is a participatory process, providing everyone with the opportunity to be heard.

## Discussion

To our knowledge, this is the first study to use concept mapping to collect diverse perspectives to define social justice. Fifty-one percent of the respondents were Black/African American people, an audience of research interest. The study identified five unique, but related, social justice clusters. Data from this study can be used to drive the Center’s operations, types of and approaches to research, relationships, training approaches, community engagement, and practices to reduce chronic disease health disparities. Several important findings emerged from this study.

Faden and Powers state that social justice is a process, goal, and the foundational moral justification for public health.^17^ Statements within the clusters represented not only processes and goals but also actions, behaviors, principles, attitudes, and norms. For example, the Empathy, Awareness, and Understanding cluster was inclusive of actions, attitudes, values, knowledge, and beliefs. The Education and Systems Change and Policy Design and Implementation clusters were inclusion of macro-level actions that demonstrated social justice. Equity and Leveling the Playing Field and Access to Services and Fair Living Standard clusters represented action steps that required individual or systems level change to assure equal opportunities. The results suggested that social justice processes include three critical steps: awareness, acknowledgment, and actions.

Our social justice clusters were consistent with the six essential dimensions of well-being (health, personal security, reasoning, respect, attachment, and self-determination) identified by Faden and Powers.^17^ For example, access to health care emerged within several clusters: “providing protection to those who are vulnerable” represented the idea of personal security, “the ability to vote without prejudice” represented reasoning, “valuing others and treating them with love” represented respect and attachment, and “equal applications of rules to everyone” represented self-determination. Our definitions were also consistent with other social justice definitions.^29,44^ That is, prior definitions have defined social justice as a fundamental human right, a moral obligation demonstrated by action, equal distribution of benefits and burdens in society,^11,13,28^ and minimizing vulnerability and social hierarchies.^45^

High mean cluster ratings for all the social justice thematic clusters suggest that all aspects of social justice were deemed important. This may be because of the high percent of Black/African Americans or women participants who historically have lived experiences of health disparities and social and structural barriers. These data have informed actions within our Center. Because all social justice statements/principles identified in the study were rated highly, mean cluster and statement ratings provided limited utility for prioritizing the most important social justice principles. In addition, some social justice principles identified in the study are out of the scope of our Center (e.g., “Punishing police brutality more harshly”). Therefore, we chose to focus on prioritizing the most actionable social justice constructs and principles identified in the participant statements by convening an *ad hoc* working group to discuss actionable social justice statements within the Center. This team first examined the action steps that the Center was already taking toward advancing social justice and then developed additional strategies to advance social justice based on the statements generated in the current study. These investigators and research staff continue to meet and work with the evaluation team to determine the Center’s progress in meeting the social justice advancement objectives. Some examples of actions taken in the Center are as follows: Staff-led monthly in-service trainings help build critical research and technical skills on topics including implementing service projects in the community, learning how to create presentations, and film discussions that focus on racial injustice. These trainings were developed and informed by the statements, “Becoming cognizant of our own contributions to social injustices,” “Taking responsibility for the welfare of all residents of the state and not just our own welfare,” and “All people are able to fulfill their full potential.” The statement, “Social justice is participatory and involves everyone having equal opportunity to be heard,” led to the Center holding annual community–academic partner multiday leadership workshops that led to an increased focus on food security and transportation equity in 2023 and 2024. The Center community advisory board is compensated at the same rate as the scientific advisory board, so as not to privilege one group, which related directly to the statement, “Everyone can earn a fair and livable wage.” The Social Justice Coalition, after the current study, recognizes that there is a need for “grassroots hours to ensure the community can trust and relate to frameworks designed to meet a shared and desired outcome.” The Social Justice Coalition also focuses on “true inclusion and access to improve the lives of all people, especially vulnerable populations and communities.” Post-study, as part of the evaluation process, Center members report ways in which they have incorporated social justice principles into the team’s work. These are just several examples of actions taken with the Center to promote social justice.

This study had several limitations. The sample was limited to Center affiliates, and we are not positioned to act on all perspectives. More than half of the participants were Black/African American, representing an audience of interest. We did not ask about rural residency or chronic disease status. Although it is possible that not all social justice perspectives were represented, we are confident that the 52 statements represent the most comprehensive definitions collected to date. Approximately 45% of invited eligible Center affiliates participated in the study, yet we received rich data to inform our strategic planning process. Additional rating prompts, such as rating the feasibility of implementing the social justice principles or actions, may have provided complementary information to the rating that evaluated the importance of the social justice statements. Due to the limited feasibility of convening all study participants, we did not get input from all participants for the interpretation phase. However, study results were presented at a community health impact conference breakout group that solicited thoughts and feedback on the final operationalization of social justice.

## Conclusions and Health Equity Implications

This study provided data on how members of our Center conceptualized social justice, which may be informative to understanding the complex societal structures, beliefs, and values that can be targeted to promote social justice and reduce health disparities. That is, using a broad focus prompt, participants affiliated with the Center engaged actively in identifying key principles for promoting social justice at multiple levels in the NIMHD Minority Health and Health Disparities Research Framework.^2^ Social justice approaches must focus on processes and actions at the individual, interpersonal, community, and societal levels that focus on changing behaviors, the physical/built and socio-cultural environment, and health care systems. In rural geographic locations such as Arkansas, health ranks among the lowest in the nation,^46^ whereas social and structural disparities rank among the highest. Nothing short of a social justice approach that is diffused throughout multiple organizations will help to ameliorate the endemic challenges now complicated by COVID-19 pandemic, inflation, political turmoil, and growing economic disparities. Studies are needed to define the metrics for how social justice is achieved in research ecosystems, where absence impacts the workforce and health equity for all marginalized communities.

## Abbreviations Used

COVID-19: 2019 novel Coronavirus
M: mean
NIMHD: National Institute on Minority Health and Health Disparities
SD: Standard Deviation
US: United States
